# Supplementary material for: Wake-like skin patterning and neural activity during octopus sleep
Source: Nature. 2023 Jun 28;619(7968):129–34. doi: 10.1038/s41586-023-06203-4 (PMC10322707; doi:10.1038/s41586-023-06203-4)
Supplement: Supplementary file 1 — Reporting Summary [file 41586_2023_6203_MOESM1_ESM.pdf]

nature portfolio

Corresponding author(s): Sam Reiter

Last updated by author(s): 03/10/2023

Reporting Summary

Nature Portfolio wishes to improve the reproducibility of the work that we publish. This form provides structure for consistency and transparency in reporting. For further information on Nature Portfolio policies, see our Editorial Policies and the Editorial Policy Checklist.

Statistics

For all statistical analyses, confirm that the following items are present in the figure legend, table legend, main text, or Methods section.

n/a	Confirmed	
☐	☒	The exact sample size (*n*) for each experimental group/condition, given as a discrete number and unit of measurement
☐	☒	A statement on whether measurements were taken from distinct samples or whether the same sample was measured repeatedly
☐	☒	The statistical test(s) used AND whether they are one- or two-sided<br>*Only common tests should be described solely by name; describe more complex techniques in the Methods section.*
☐	☒	A description of all covariates tested
☐	☒	A description of any assumptions or corrections, such as tests of normality and adjustment for multiple comparisons
☐	☒	A full description of the statistical parameters including central tendency (e.g. means) or other basic estimates (e.g. regression coefficient) AND variation (e.g. standard deviation) or associated estimates of uncertainty (e.g. confidence intervals)
☐	☒	For null hypothesis testing, the test statistic (e.g. *F*, *t*, *r*) with confidence intervals, effect sizes, degrees of freedom and *P* value noted<br>*Give P values as exact values whenever suitable.*
☒	☐	For Bayesian analysis, information on the choice of priors and Markov chain Monte Carlo settings
☒	☐	For hierarchical and complex designs, identification of the appropriate level for tests and full reporting of outcomes
☒	☐	Estimates of effect sizes (e.g. Cohen's *d*, Pearson's *r*), indicating how they were calculated

Our web collection on statistics for biologists contains articles on many of the points above.

Software and code

Policy information about availability of computer code

Data collection	Video data was collected using PylonRecorder2:0.6, a C++ application making use of the Basler Pylon5 SDK. Electrophysiological data was collected using SpikeGLX 3.0., with camera synchronization achieved using an Arduino to activate camera frame exposure and to register TTL times. Light sheet microscopy was performed using custom software written in LabVIEW 2019.
Data analysis	Video clips were cut using FFMPEG:3.4.11. Core video analysis was performed using scripts written in Python:3.6 and 3.7. Electrophysiological analysis and further processing of video data was done with Matlab:2019a. Spectra were calculated using the Chronux toolbox:2.12. Segmentation was done using the Detectron2 platform:0.1.3. Neural network analysis was done using Keras, included in tensorflow 2.0. Microscopy data was analyzed using ImageJ:1.53, Imaris:9.7.1, and Slicer:4.1.1

For manuscripts utilizing custom algorithms or software that are central to the research but not yet described in published literature, software must be made available to editors and reviewers. We strongly encourage code deposition in a community repository (e.g. GitHub). See the Nature Portfolio guidelines for submitting code & software for further information.

Data

Policy information about availability of data

All manuscripts must include a data availability statement. This statement should provide the following information, where applicable:

- Accession codes, unique identifiers, or web links for publicly available datasets
- A description of any restrictions on data availability
- For clinical datasets or third party data, please ensure that the statement adheres to our policy

Data are available from the corresponding author on request. A small dataset is provided with the analysis code for demonstration purposes. Data is available at https://github.com/oist/pophale2023. The pretrained VGG19 network used in this study is included in Tensorflow 2.0, available at tensorflow.org.

Human research participants

Policy information about studies involving human research participants and Sex and Gender in Research.

Reporting on sex and gender	n/a
Population characteristics	n/a
Recruitment	n/a
Ethics oversight	n/a

Note that full information on the approval of the study protocol must also be provided in the manuscript.

Field-specific reporting

Please select the one below that is the best fit for your research. If you are not sure, read the appropriate sections before making your selection.

☒ Life sciences ☐ Behavioural & social sciences ☐ Ecological, evolutionary & environmental sciences

For a reference copy of the document with all sections, see nature.com/documents/nr-reporting-summary-flat.pdf

Life sciences study design

All studies must disclose on these points even when the disclosure is negative.

Sample size	No statistical methods were used to predetermine sample sizes. The number of octopus per for behavioral experiments was determined by seeking to match or exceed that of previous publications on cephalopod resting behavior (refs. 6, 16,17). For electrophysiological experiments we sought to maximize the number of recorded animals within the season of octopus availability (wild caught animals).
Data exclusions	Animals were excluded from further behavioral analysis if they exhibited signs of stress after several days of being moved into experimental filming tanks.
Replication	Experiments reported in this study followed pilot experiments establishing methods for octopus behavioral filming and electrophysiology. Once methodology and instrumentation was established, all replication attempts were successful. Experiments were performed independently multiple times, sample sizes are stated throughout the manuscript.
Randomization	The experiments in our study were mostly observational in nature, and did not involve experimental groups. Manipulation experiments used the same animal before/after manipulation as controls. Randomization was thus not relevant to our study.
Blinding	Experiments and data analysis were not performed blind to experimental conditions. Most experiments in our study consist of analysis of a biological system without experimental and control groups. Manipulation experiments largely relied on automated analysis comparing times before and after experimental treatment. Active bouts were identified manually using a quantitative readout of skin brightness and video confirmation. In all cases blinding was not relevant to our study.

Reporting for specific materials, systems and methods

We require information from authors about some types of materials, experimental systems and methods used in many studies. Here, indicate whether each material, system or method listed is relevant to your study. If you are not sure if a list item applies to your research, read the appropriate section before selecting a response.

Materials & experimental systems

n/a	Involved in the study
☒	☐ Antibodies
☒	☐ Eukaryotic cell lines
☒	☐ Palaeontology and archaeology
☐	☒ Animals and other organisms
☒	☐ Clinical data
☒	☐ Dual use research of concern

Methods

n/a	Involved in the study
☒	☐ ChIP-seq
☒	☐ Flow cytometry
☒	☐ MRI-based neuroimaging

Animals and other research organisms

Policy information about studies involving animals; ARRIVE guidelines recommended for reporting animal research, and Sex and Gender in Research

Laboratory animals	This study did not involve laboratory animals.
Wild animals	Octopus laqueus of unknown sex and age were captured from tidal pools in Okinawa, Japan. Animals were transported to the OIST marine station/main campus in transport tanks. Transport took ~15 minutes. Following behavioral observation, animals were either used in terminal experiments and euthanized under deep anesthesia before brain extraction and analysis, or died following natural senescence. Animals were not released back to the wild.
Reporting on sex	Our study used both male and female animals (16 females, 13 males). Active sleep behavior was observed in all octopuses, motivating us to pool data across sexes. Sex was not considered further in the study design. Sex was determined through examination of arms (presence of hectocotylus in males) and/or the presence of eggs in the abdomen of females.
Field-collected samples	This study did not involve samples collected from the field
Ethics oversight	The experiments in this study were approved by the OIST Animal Care and Use Committee under approval numbers 2019-244-6 and 2022-364.

Note that full information on the approval of the study protocol must also be provided in the manuscript.
